# GL-VSLAM: A General Lightweight Visual SLAM Approach for RGB-D and Stereo Cameras

**DOI:** 10.3390/s25247467

**Published:** 2025-12-08

**Authors:** Xu Li, Tuanjie Li, Yulin Zhang, Ziang Li, Lixiang Ban, Yuming Ning

**Affiliations:** 1 College of Information Engineering, Tarim University, Alar 843300, China; 2 School of Mechano-Electronic Engineering, Xidian University, Xi’an 710071, China

**Keywords:** simultaneous localization and mapping, optical flow, feature detection, uniform motion model, keypoint matchings

## Abstract

**Highlights:**

- A general lightweight SLAM system is proposed that achieves high real-time performance by replacing traditional feature matching with efficient sparse optical flow tracking.
- A coarse-to-fine pose estimation strategy ensures robust and accurate camera localization through RANSAC PnP and subsequent nonlinear optimization.

**What are the main findings?**

- A lightweight visual SLAM approach for RGB-D and stereo cameras is proposed, incorporating loop detection and re-localization.
- A three-strategy adaptive ORB feature extraction method is combined with a coarse-to-fine, two-stage pose estimation process to improve localization accuracy.

**What are the implications of the main findings?**

- The findings demonstrate that high-precision visual localization and mapping can be achieved without compromising computational efficiency.
- The integration of multi-level optical flow with coarse-to-fine pose refinement enables GL-VSLAM to balance accuracy and speed for deployment on resource-limited platforms.

**Abstract:**

Feature-based indirect SLAM is more robust than direct SLAM; however, feature extraction and descriptor computation are time-consuming. In this paper, we propose GL-VSLAM, a general lightweight visual SLAM approach designed for RGB-D and stereo cameras. GL-VSLAM utilizes sparse optical flow matching based on uniform motion model prediction to establish keypoint correspondences between consecutive frames, rather than relying on descriptor-based feature matching, thereby achieving high real-time performance. To enhance positioning accuracy, we adopt a coarse-to-fine strategy for pose estimation in two stages. In the first stage, the initial camera pose is estimated using RANSAC PnP based on robust keypoint correspondences from sparse optical flow. In the second stage, the camera pose is further refined by minimizing the reprojection error. Keypoints and descriptors are extracted from keyframes for backend optimization and loop closure detection. We evaluate our system on the TUM and KITTI datasets, as well as in a real-world environment, and compare it with several state-of-the-art methods. Experimental results demonstrate that our method achieves comparable positioning accuracy, while its efficiency is up to twice that of ORB-SLAM2.

## 1. Introduction

Simultaneous localization and mapping (SLAM) encompasses techniques aimed at concurrently constructing a map of an unfamiliar environment and determining the sensor’s position within it. Emphasizing real-time performance, SLAM plays a vital role in enabling autonomous vehicles, drones, and mobile robots to navigate and localize effectively in uncharted areas [1,2,3]. Among various sensors, cameras are particularly advantageous due to their compact size, low energy consumption, and ability to capture rich information, making them one of the key external sensors for SLAM systems. When cameras are used as the main sensor, the system is referred to as visual SLAM (VSLAM). VSLAM can be divided into two main categories: direct methods, which are based on photometric measurements, and indirect methods, which rely on feature extraction and matching, depending on the visual measurement techniques and error models used. Direct methods [4,5,6] bypass the need for keypoint and descriptor extraction, instead utilizing pixel-level intensity values for matching and optimization. These methods estimate camera poses by minimizing photometric errors, which significantly enhances real-time performance. Direct methods, however, face challenges in map reuse, loop detection, and re-localization after tracking loss. In contrast, indirect methods [7,8] offer stable performance, are less affected by lighting conditions, and are more suitable for loop detection and map reuse. As a result, indirect methods are more widely applicable. These methods typically involve extracting sparse, stable keypoints from each frame, performing feature matching based on their descriptors, and optimizing camera poses by minimizing reprojection errors.

VSLAM requires both high accuracy and low computational cost [9,10,11]. The efficiency of feature extraction and the precision of pose estimation are key factors influencing system performance. Among various feature extraction methods [12,13,14,15], oriented FAST and rotated BRIEF (ORB) [15] features are commonly used due to their stability and efficiency. The ORB algorithm addresses the non-directional limitation of the features from the accelerated segment test (FAST) [14] corner detector and utilizes the fast-binary descriptor binary robust independent elementary features (BRIEF) [16], significantly accelerating the feature extraction process. This improves real-time performance and enhances algorithm robustness. However, the extracted feature points tend to cluster together. In situations where the sensor is obstructed or external factors cause abrupt lighting changes, images may become blurred, making it challenging to extract stable feature points using a fixed threshold. Mur-Artal [8] et al. introduced a quadtree-based ORB feature extraction method in ORB-SLAM2 to improve uniformity. However, this approach uses a fixed threshold for the entire image, which struggles to adapt to varying regional feature complexity. Additionally, the quadtree method increases computational complexity. Wu [17] et al. developed an adaptive ORB feature detection method with a variable extraction radius to enhance efficiency. However, this approach does not ensure uniform distribution or distinctiveness of feature points across different image regions. Ma [18] et al. proposed a threshold extraction algorithm based on gray clustering, offering some adaptability to ambient light changes. However, its performance is weaker at cluster boundaries, and the method incurs high computational costs.

Minimizing photometric errors in direct methods or reprojection errors in indirect methods can be framed as a nonlinear least squares problem, typically solved using bundle adjustment (BA) algorithms [19] for pose estimation and optimization. Forster et al. [5] introduced semi-direct visual odometry (SVO), which minimizes the photometric error between feature points in the previous frame and corresponding projected points in the current frame. The camera pose relative to the previous frame was then obtained, followed by optimization of the 2D coordinates of the projected points to achieve accurate pose estimation through reprojection error minimization. Direct sparse odometry (DSO) [6], proposed by Engel, is a direct visual odometry method that lacks loop detection and map reuse capabilities. It samples pixels with significant intensity gradients and incorporates photometric error and model parameters into the optimization function, enabling semi-dense mapping on a standard CPU. ORB-SLAM [7] is a real-time monocular SLAM system that utilizes feature points, capable of functioning effectively in both indoor and outdoor settings. It demonstrates strong robustness against abrupt motion. The backend utilizes bundle adjustment (BA) for global pose optimization and a loop detection algorithm based on the bag-of-words model. ORB-SLAM2 [8] extends ORB-SLAM by incorporating stereo and RGB-D cameras for sparse 3D reconstruction. ORB-SLAM3 [20] builds upon ORB-SLAM2 by integrating an inertial measurement unit (IMU). Visual–inertial navigation system—Mono (VINS-Mono) [21], a visual–inertial odometry system, employs tightly coupled sliding window nonlinear optimization, using optical flow and Harris corner points in the frontend for visual processing. The backend performs system state updates and optimizations by minimizing reprojection errors and IMU measurement residuals and supports online extrinsic calibration, loop detection, and pose graph optimization. Qin et al. later expanded VINS-Mono to develop a visual–inertial navigation system—fusion (VINS-Fusion) [22], which integrates multiple visual–inertial sensors for autonomous precise positioning.

In recent years, learning-based SLAM methods have emerged, leveraging task-specific datasets to enhance their predictive capabilities. iMAP [23] pioneered the use of a multilayer perceptron (MLP) to represent scene maps within SLAM, integrating implicit neural networks to achieve commendable tracking and mapping performance on benchmark datasets. NICE-SLAM [24] combines neural implicit decoders with hierarchical grid-based representations, facilitating scalable and efficient mapping of large-scale indoor environments. However, these methods typically require substantial computational resources and suffer from limited generalization across diverse environments.

**Figure 1:**
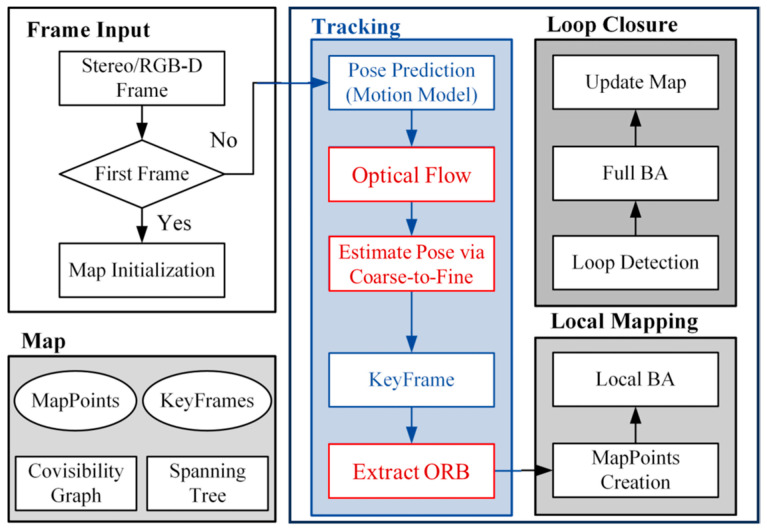
The system consists of three threads: tracking, local mapping, and loop closure. The tracking thread outputs camera pose estimates and selects keyframes in real-time. The local mapping thread performs local BA on keyframes to optimize the local map. The loop closure thread performs loop detection to correct accumulated drift and executes BA to optimize the global trajectory and map. The system maintains a map structure, including map points, keyframes, a co-visibility graph, and a spanning tree. The structure is compact and can timely remove unused information while retaining useful observations, avoiding redundant computation.

A complete VSLAM system consists of three main components: tracking, local mapping, and loop closure. The tracking thread, responsible for estimating the current camera pose in real-time, feeds this information to the backend for optimization and loop closure. Consequently, the performance and accuracy of the tracking thread are crucial for the overall efficiency of the VSLAM system. In this paper, we present a lightweight visual SLAM approach built upon ORB-SLAM2 [8]. As illustrated in Figure 1, our method leverages sparse optical flow for tracking adjacent frames within the tracking thread, eliminating the need for redundant feature extraction, which significantly enhances the system’s real-time performance.

The main contributions are as follows:

(1) We propose a lightweight visual SLAM approach for RGB-D and stereo cameras, which includes loop detection and re-localization. This directly addresses the computational bottleneck of descriptor-based feature matching in systems like ORB-SLAM2. Instead of relying on redundant feature extraction, correspondences between adjacent frames are established by minimizing grayscale error. Keypoints are only extracted, and descriptors are computed to build a bag-of-words model [25] for backend optimization and loop closure detection when a keyframe decision is made.
(2) We propose a three-strategy ORB feature adaptive extraction algorithm to overcome the poor adaptability of fixed-threshold feature extraction to varying environments. During feature extraction, an adaptive threshold is used to retain real features while considering the difference between global and local images, improving adaptability to complex environments. When using quadtrees to filter feature points, the maximum depth of the quadtree is set to avoid over-subdivision while ensuring the number of extracted feature points, thus improving algorithm efficiency.
(3) We introduce a coarse-to-fine strategy to enhance pose estimation accuracy in two stages, effectively resolving the vulnerability of single-stage pose estimation in rapid motion scenarios. Initially, the pose predicted by the uniform motion model serves as the starting point for optical flow tracking, minimizing the algorithm’s iterations. Then, the camera pose estimated using PnP based on the feature points tracked by optical flow is refined by further optimizing the pose through reprojection error minimization.

The paper is structured as follows: Section 1 covers related work and introduces our system, Section 2 and Section 3 provide a detailed description of our approach, Section 4 presents the evaluation results, and Section 5 concludes the paper.

## 2. Three-Strategy ORB Feature Adaptive Extraction Algorithm

**Figure 2:**
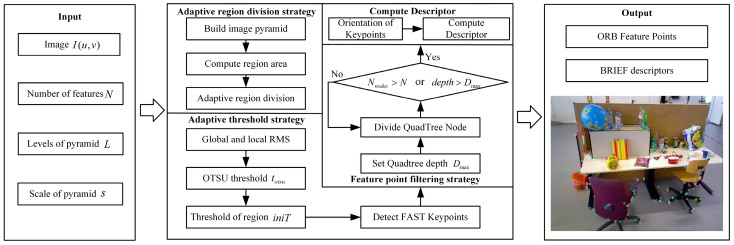
The extraction algorithm includes three strategies: adaptive region division strategy, adaptive extraction threshold strategy, and feature point filtering strategy based on an improved quadtree.

ORB-SLAM2 [8] uses a quadtree-based ORB feature extraction method to enhance uniformity. However, it applies a fixed threshold across the entire image, which does not effectively accommodate the varying complexity of features in different regions. Additionally, the introduction of the quadtree algorithm increases computational overhead. This paper proposes a three-strategy ORB feature adaptive extraction algorithm to address the above issues. Figure 2 shows the workflow diagram, where N represents the total number of quadtree nodes and “depth” represents the current quadtree depth. The main implementations are as follows:

(1) Adaptive region division strategy adaptively divides regions based on the area and number of extracted features in each pyramid level, improving the rationality of region division and adaptability to complex environments.
(2) Adaptive extraction threshold strategy adaptively adjusts the threshold through gray information, global threshold extraction coefficient, and the OTSU [26] threshold or minimum extraction threshold of each region in each level image. This can maximally retain real features while considering differences between global and local images, better adapting to changes in brightness and textures of different regions. This strategy enhances the accuracy and robustness of feature extraction, improving the precision of feature matching.
(3) The feature point filtering strategy based on an improved quadtree adjusts the maximum division depth of quadtree nodes at different pyramid levels. This approach not only ensures the desired number of feature points but also eliminates redundant points, retaining those with higher responses. As a result, the spatial distribution of feature points is optimized, enhancing the overall efficiency of the algorithm.

### 2.1. Adaptive Region Division Strategy

**Figure 3:**
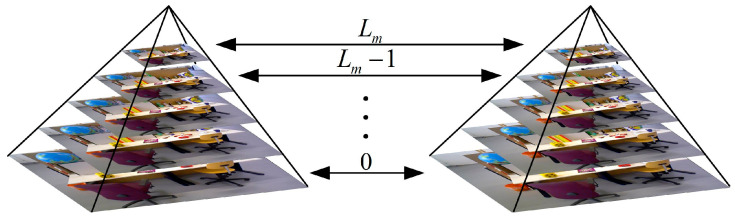
Pyramids built based on the level and scale factor.

The image pyramid is built as shown in Figure 3. The number of feature points is distributed to each level of the pyramid, with $N_{k}$ as the number of points extracted in the kth level:

(1) $$N_{k}=N(1-s^{-2}){\left(s^{-2}\right)}^{k}/(1-{\left(s^{-2}\right)}^{L})$$

where N is the expected number of feature points, s is the pyramid scale factor, and L is the number of pyramid levels. Experimental tests found that by replacing $s^{-2}$ with $s^{-1}$ in the above formula, it can increase the number of feature points in higher levels and make the distribution of feature quantities more uniform across levels, improving the precision of feature matching. According to the size of each level image and the allocated number of feature points $N_{k}$, the side length of the regions to be divided in the kth level image can be calculated. Let the width and height of the kth level image be $W_{k}$ and $H_{k}$, respectively. Then,

(2) $$S=round(\sqrt{\lambda \times W_{k}\times H_{k}/N_{k}})$$

where S represents the length of the side of the divided region. The round() function takes the integer of the function result, and λ is a proportional coefficient. The number of columns C and rows R of the regions for each pyramid level are then calculated based on the size of the corresponding level image as follows:

(3) $$C=W_{k}/S,h_{k}=round(H_{k}/R)$$

The actual width $w_{k}$ and height $h_{k}$ of the regions are then determined based on the calculated number of C columns and rows R as follows:

(4) $$w_{k}=round(W_{k}/C),h_{k}=round(H_{k}/R)$$

In our implementation, the expected number of features N is set to 1000, the pyramid scale factor s to 1.2, and the number of pyramid levels L to 8, following the parameter configuration recommended in the original ORB algorithm publication [8]. The proportional coefficient k in Equation (2) is empirically set to 0.8 to balance the granularity of region division and computational efficiency.

### 2.2. Adaptive Extraction Threshold Strategy

ORB features are derived from FAST corners, with additional scale and rotation invariance. FAST corners identify keypoints by comparing the grayscale differences between a pixel and its neighbors. The feature extraction threshold defines the minimum grayscale difference necessary to detect a corner, which also represents the maximum noise tolerance the method can handle. This paper adaptively adjusts the extraction threshold based on the root mean square contrast divided into global and local regions. This can better adapt to changes in brightness and textures of different regions, thereby enhancing the accuracy and robustness of feature extraction. The image is divided into L pixel levels. $n_{i}$ represents the number of pixels, and $p_{i}$ represents the probability at the pixel level. The threshold t divides the image into foreground $C_{1}$ and background $C_{2}$. Then,

(5) $$p_{i}=n_{i}/N,\;\sum_{i=1}^{L}n_{i}=N,\;\sum_{i=1}^{L}p_{i}=1$$

(6) $$P_{1}(t)=\sum_{i=1}^{t}p_{i},P_{2}(t)=\sum_{i=t+1}^{L}p_{i}=1-P_{1}(t)$$

where $P_{1}(t)$ and $P_{2}(t)$ represent the probability that a pixel is assigned to the foreground and the background, respectively. Then, the foreground gray mean ${\bar{I}}_{1}$ and background gray mean ${\bar{I}}_{2}$ are as follows:

(7) $${\bar{I}}_{1}=\sum_{i=1}^{t}iP(i|C_{1})=\frac{1}{P_{1}(t)}\sum_{i=1}^{t}ip_{i}$$

(8) $${\bar{I}}_{2}=\sum_{i=t+1}^{L}iP(i|C_{2})=\frac{1}{P_{2}(t)}\sum_{i=t+1}^{L}ip_{i}$$

The between-class variance is then obtained:

(9) $$\omega (t)=P_{1}P_{2}{\left({\bar{I}}_{1}-{\bar{I}}_{2}\right)}^{2}$$

The L pixel levels are traversed in the region to obtain the gray threshold $t_{otsu}$, which maximizes the between-class variance ω(t). According to the contrast and gray distribution characteristics of the image, a self-adaptive threshold is dynamically calculated for FAST corner detection:

(10) $$T=\varepsilon \times (\frac{1}{n}\sum_{i=1}^{n}I_{\max}^{i}-\frac{1}{n}\sum_{i=1}^{n}I_{\min}^{i})$$

where ε is a proportional coefficient. $I_{\max}^{i}$ and $I_{\min}^{i}$ represent the maximum and minimum gray values in the image, respectively. In our implementation, the coefficient ε is set to 5 (or 10 in high-contrast scenes), following the standard configuration of the FAST corner detector. To suppress noise-induced responses, the minimum extraction threshold δ  is fixed at 5. These parameter settings were selected based on empirical evaluation on the TUM [27] and KITTI [28] datasets and are consistent with commonly used FAST/ORB configurations.

The root mean square (RMS) contrast of an image can reflect the degree of dispersion between the pixel values and the mean value. A larger value indicates clearer edge information and better image quality, requiring a larger threshold extraction coefficient. Conversely, when the RMS is small, a smaller threshold extraction coefficient should be set. Firstly, the RMS contrast of each local divided region is calculated. If the value is greater than the preset threshold, the feature extraction threshold of the region is calculated directly according to Equation (10). Otherwise, the RMS of the whole image is calculated first to determine the threshold extraction coefficient, and then the region-adaptive threshold is calculated by combining the brightness of the region so as to fully consider the quality differences between different regions of the image, realize automatic adjustment of the threshold, and improve the corner detection effect, as shown in Equation (12).

(11) $$iniT=\left\{\begin{array}{c} T,\;\;\sigma^{cell}>\alpha \\ T^{\prime },\;\;\sigma^{cell}\leq \alpha \end{array}\right.$$

(12) $$T^{\prime }=\left\{\begin{array}{cc} \delta \times t_{otsu} & \bar{I}(u,v)>\beta \\ \delta \times T_{min} & \bar{I}(u,v)\leq \beta \end{array}\right.,\;\delta =\left\{\begin{array}{cc} 0.2 & \sigma^{total}>\alpha \\ 0.1 & \sigma^{total}\leq \alpha \end{array}\right.$$

where $\sigma^{cell}$ and $\sigma^{total}$ represents the global and the local RMS contrast, δ represents the global threshold extraction coefficient, α=30 represents the adaptive parameter of image quality, and β=80 represents the adaptive parameter of foreground and background segmentation threshold.

### 2.3. Feature Point Filtering Strategy Based on Improved Quadtree

In the feature point filtering process of the quadtree division method in ORB-SLAM2 [8], the splitting depth of the quadtree is not restricted, resulting in too many splits and reducing the efficiency of the algorithm. In addition, the extracted feature points are overly uniformly distributed. To solve the above problems, this paper dynamically sets the maximum depth of quadtree nodes based on the number $N_{k}$ of feature points to be extracted on the kth layer of the pyramid. The minimum D when $4^{D}>N_{k}$ is taken as the depth of the kth layer image, and the maximum depth of this layer image is calculated as follows:

(13) $$D_{\max}=D+i$$

where i represents the number of child nodes without feature points among the 4 child nodes after splitting the root node of the quadtree, 0≤i<4. First, each layer image is taken as the root node of the quadtree and split into 4 child nodes. Then, it is judged whether each child node contains feature points. If yes, the feature point within the child node is retained when each child node contains only one feature point. When each child node contains multiple feature points, the child node is split into 4 child nodes, and the depth d and the total number of nodes $N_{nodes}$ after splitting are recorded; otherwise, the child node is deleted. When the total number of nodes $N_{nodes}$ in each layer image satisfies $N_{nodes}>N_{k}$ or d satisfies $d>D_{\max}$ or all nodes inside only have one feature point left, the feature point with the maximum Harris response in all nodes is taken as the screened feature point.

This can avoid excessive redundant subdivision while ensuring the number of extracted feature points, thus improving the efficiency of the algorithm. At the same time, by limiting the depth to control the number of feature points in each node, it can make the distribution of feature points in different regions of the image more uniform. The pseudocode of the three-strategy ORB feature adaptive extraction algorithm is shown in Algorithm 1, where KpsNum represents the number of feature points in the node, $N_{nodes}$ represents the number of nodes, and $SubN_{nodes}$ represents the number of sub-nodes of the current node.
**Algorithm 1**: Three-strategy ORB feature adaptive extraction algorithm**Input**: Image $I^{total}$, Number of features N, Levels of pyramid L, Scale factor of pyramid s**Output**: Uniformly distribute N feature points and descriptors1: Compute Pyramid($I^{total}$, s, L);2: **for** k←1 to L **do**3:  Divide image into R×C cells;4:  **for** i←1 to R **do**5:     **for**  j←1 to C **do**6:       $I^{cell}\leftarrow I^{total}[i][j]$;7:       Compute Adaptive threshold iniT;8:       KeyPoints← Feature Extraction($I^{cell}$, iniT);9:     **end for**10:  **end for**11:  Set maximum depth $D_{\max}$ of quadtree by;12:  **while** $d<D_{\max}$ **do**13:    **if**  KpsNum>0 **then**14:      **if**  KpsNum>1 **then**15:        Split Node;16:      **else**17:        Save Node;18:      **end if**19:    **end if**20:    **if** $N_{nodes}>N_{k}$ **or** $N_{nodes}=SubN_{nodes}$ **then**21:      Finish Split and Non-Maximum Suppression;22:    **end if**23:  **end while**24:  Restore the feature points to original image;25: **end for**26: **return** N feature points and descriptors;

To ensure the reproducibility of our method, all hyperparameters used in the three-strategy ORB feature extraction algorithm and the coarse-to-fine pose estimation pipeline are summarized in Table 1, together with the rationale behind their selection. These parameters follow the standard ORB-SLAM2 [8] configuration or are empirically tuned to balance feature density, robustness, and computational efficiency.

## 3. Coarse-to-Fine Pose Estimation

Our Tracking thread adopts a uniform motion model to predict the current camera pose, then projects the 3D points in the world coordinate system to 2D points in the pixel coordinate system of the current frame based on the predicted pose, providing initial values for optical flow tracking. We use a multilayer optical flow method based on image pyramids to track feature points and remove tracking outlier pairs through backward optical flow tracking and the RANSAC algorithm. Finally, the camera pose estimated by PnP is used as the initial value, and the accurate camera pose is obtained by minimizing the reprojection error, as shown in the pseudocode in Algorithm 2.
**Algorithm 2**: Coarse-to-fine pose estimation algorithm**Input**: Keypoints p and world points $P_{w}$ on the reference frame; last two frame poses $T_{lw}$, $T_{cw}$;**Output**: Optimized camera pose $T_{nw}$;1: Predict keypoints according to the Uniform Motion Model by Equation (16): $q(x^{*},y^{*})$;2: Solve the movement vector d with Algorithm 3;3: Establish keypoint correspondences between adjacent frames by refining inliers using the outlier removal strategy described in Section 3.2;4: Calculate the initial camera pose using RANSAC PnP on the inliers;5: Further refine the pose by optimizing Equation (22);6: **return** $T_{nw}$;

**Algorithm 3**: Multi-level optical flow method based on the uniform motion model**Input**: Current frame $I_{c}$ and keypoint p(x,y), next frame $I_{n}$, and initial value $q(x^{*},y^{*})$;**Output**: Movement vector d=[dx,dy] and keypoint correspondence;1: Build image pyramid: $\{I_{l}^{L}\},{\left\{I_{c}^{L}\right\}}_{L=0,\ldots ,L_{m}}$;2: Keypoints on pyramid level L: $p^{L}\;=\;p\;/\;s^{L}$;3:  **for** $L=L_{m},L_{m}-1,\ldots ,0$ **do**4:   Objective function by Equation (21): $\underset{d^{L}}{\min}\;F^{L}(d^{L})$;5:   Iteratively compute the optical flow of level L: $d^{L}$;6:   Use the optical flow result of level L as the initial value for optical flow computation of level L−1: $q^{L-1}=sd^{L}$;7:  **end for**8: **return**
$d=d^{0}$ and $I_{c}(x,y)\to I_{n}(x+dx,y+dy)$;

### 3.1. Multi-Level Optical Flow Method Based on the Uniform Motion Model

**Figure 4:**
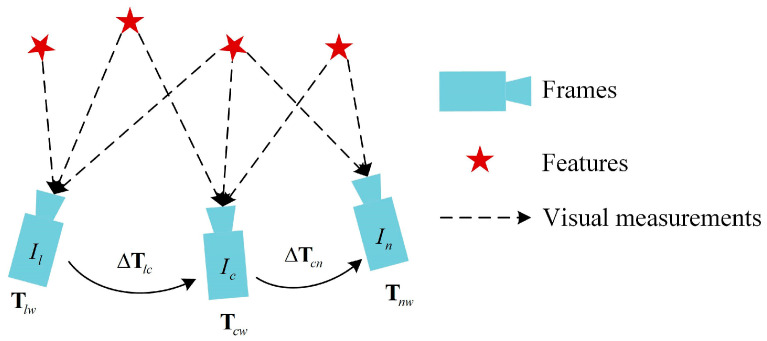
Uniform motion model. $I_{l},I_{c},I_{n}$ are three consecutive frames, $T_{lw},T_{cw},T_{nw}$ represent the poses of the three image frames in the world coordinate system, and $\Delta T_{lk},\Delta T_{cn}$ represent the relative motion increments between adjacent frames.

Uniform Motion Model: It is assumed that the camera’s linear and angular velocities remain unchanged from the previous frame to the current frame. By calculating the relative motion increment between the two frames based on velocity and time interval, the current frame pose is estimated based on the previous frame pose using the estimated motion increment. The uniform motion model is shown in Figure 4, from which we can obtain the following:

(14) $$\Delta T_{cn}=T_{nw}T_{cw}^{-1},\;\Delta T_{lc}=T_{cw}T_{lw}^{-1}$$

(15) $$T_{nw}=T_{cw}T_{lw}^{-1}T_{cw}$$

Given the poses of two frames $I_{l}$, $I_{c}$ in the world coordinate system and the homogeneous coordinate $P_{w}(X,Y,Z,1)$ of a 3D point in the world coordinate system, the predicted homogeneous coordinate $q^{\prime }(x^{*},y^{*},1)$ of the 2D point in the pixel coordinate system of the frame $I_{n}$ is as follows:

(16) $$q^{\prime }=\frac{1}{Z}KT_{nw}P_{w}$$

where K represents the camera intrinsic matrix.

Optical flow tracks the movement of pixels over time in image sequences using the correlation between consecutive frames to establish correspondences. The principles of optical flow are based on two assumptions [29]:

Grayscale invariance: The pixel grayscale value of the same spatial point is the same in different images. Assuming a spatial point at time t is located at position (x,y) in the previous frame image with grayscale value I(x,y,t), at time t+dt, the spatial point is located at position (x+dx,y+dy) in the current frame, based on the grayscale invariance assumption.

(17) I(x+dx,y+dy,t+dt)=I(x,y,t)

Taylor expanding the left side of the equation and keeping the first-order term:

(18) $$I(x,y,t)+I_{x}dx+I_{y}dy+I_{t}dt=I(x,y,t)$$

where $I_{x}=\partial I/\partial x$ and $I_{y}=\partial I/\partial y$ represent the image gradient in the x and y directions, respectively, at the pixel point, and $I_{t}=\partial I/\partial t$ represents the grayscale derivative over time at that point. Let u=dx/dt and v=dy/dt represent the pixel motion velocities in the x and y axes, respectively, then

(19) $$\left[\begin{array}{cc} I_{x} & I_{y} \end{array}\right]\left[\begin{array}{c} u\\ v \end{array}\right]=-I_{t}$$

Equation (19) cannot obtain a unique solution for (u,v); therefore, the neighborhood motion consistency assumption is needed.

Neighborhood motion consistency: All pixels within a neighborhood have the same motion. For a ω×ω neighborhood centered at pixel (x,y), all pixels within it have consistent motion:

(20) $${\left[\begin{array}{cc} I_{x} & I_{y} \end{array}\right]}_{n}\left[\begin{array}{c} u\\ v \end{array}\right]=-I_{tn},\;n=1,\ldots ,\omega^{2}$$

Equation (20) is an overdetermined system of equations with respect to u and v, so the optical flow matching problem can be converted into solving a nonlinear optimization problem.

To find the matching point $\left(x^{*}+dx,y^{*}+dy\right)$ of a point p(x,y) in image frame $I_{c}$ in image frame $I_{n}$ based on the initial value $q(x^{*},y^{*})$ estimated by the motion model, where d=[dx,dy] is the optical flow of pixel p, the optimal pixel offset is obtained by minimizing the grayscale error with the following objective function:

(21) $$\underset{dx,dy}{\min}F(dx,dy)=\sum_{i}^{n}||I_{c}(x_{i},y_{i})-I_{n}(x_{i}^{*}+dx,y_{i}^{*}+dy)|{|}^{2}$$

Due to the grayscale invariance assumption and the neighborhood motion consistency assumption of optical flow, optical flow methods are quite sensitive to changes in image brightness. Additionally, the motion of objects between adjacent frames must be sufficiently small. Therefore, an image pyramid is introduced to address situations where the image displacement is large, as shown in Figure 3.

When the pixel motion in the original image is fast, the pixel motion speed at higher layers is progressively decreased according to the pyramid scale factor S, ensuring the optical flow assumptions by reducing pixel motion. Optical flow tracking is performed from the top layer to the bottom layer of the image pyramid sequentially, with the result of each layer used as the initial value for optical flow at the next layer, and the optical flow result at the bottom layer taken as the final result for this optical flow tracking, as shown in the pseudocode in Algorithm 3.

### 3.2. Outlier Removal

The multi-level optical flow method based on the uniform motion model can better track feature points of keyframes, but there may still be some incorrectly tracked feature points. To this end, we adopt two efficient strategies to refine inliers:

(1) Reverse optical flow tracking: Taking the optical flow obtained from tracking as the prediction value and tracking it reversely to the current frame, the distance between the feature points corresponding to the reverse tracking and forward tracking is compared; if the distance is greater than a threshold, it is considered as an outlier and removed.
(2) Fundamental matrix based on RANSAC [30]: For the feature points remaining after outlier removal by reverse optical flow tracking, the fundamental matrix between the previous frame and current frame is calculated, and RANSAC is used to remove outliers.

### 3.3. Pose Optimization

We employ a coarse-to-fine strategy to enhance pose estimation accuracy. Initially, an approximate camera pose is obtained using the RANSAC-based PnP algorithm on feature points tracked via optical flow. The pose is then refined by minimizing reprojection error. For RGB-D cameras, the 3D coordinates of feature points are directly derived from the depth image. In the case of stereo cameras, optical flow is used to match feature points between the left and right images, and their 3D positions in the world coordinate system are computed through triangulation. This approach begins with a rough pose estimation and iteratively refines it to improve accuracy.

According to feature point coordinates and their 3D positions in the world coordinate system, a least squares problem is built to optimize the camera pose $T_{cw}\in SE(3)$ so that the reprojection error between the 2D coordinates of feature points and the 2D coordinates obtained by projecting the 3D points according to the current pose is minimized, with i∈χ the set of all matches:

(22) $$T_{nw}^{*}=\underset{T_{nw}}{\arg\min}\frac{1}{2}{\sum_{i\in \chi }\left\|P_{uv}^{i}-\frac{1}{s_{i}}KT_{nw}P_{w}^{i}\right\|}_{2}^{2}$$

where $P_{uv}^{i}$ denotes the homogeneous pixel coordinate of the i-th inlier feature point in the current frame, $P_{w}^{i}$ is the corresponding 3D point in the world coordinate system, $s_{i}$ represents the depth value (or the stereo-derived scale) of the i-th feature point, and K is the intrinsic matrix of the camera. The initial pose estimate $T_{nw}$ is obtained using the RANSAC-based PnP algorithm and is subsequently refined by minimizing Equation (22) through a nonlinear least squares optimization.

### 3.4. New Keyframe Decision

By using keyframes to represent neighboring frames, the number of frames to be optimized can be effectively reduced, improving the computational efficiency of the algorithm. This paper integrates temporal description, spatial description, and scene description to determine whether to add a keyframe:

(1) The time interval between the current frame and the previous keyframe exceeds a threshold t;
(2) The number of inlier points tracked by optical flow in the current frame is less than a threshold $N_{n}$;
(3) The ratio of inlier matches between the current and previous frames falls below a predefined threshold $K_{r}$;
(4) The pose change between the current frame and the previous keyframe is greater than a threshold $T_{k}$.

## 4. Experimental Results

To ensure a fair comparison, we reproduced the results of ORB-SLAM2 [8], ORB-SLAM3 [20], and VINS-Fusion [22] using their official implementations on our evaluation platform. All methods were tested under the same hardware and dataset conditions. For learning-based methods such as NICE-SLAM [24] and iMAP [23], whose source codes involve complex training pipelines, we referred to the results reported in their original publications. The parameters for all reproduced methods were kept consistent with the original papers. Each sequence was executed five times, and the median results are reported to reduce the effects of runtime variability.

For both RGB-D and stereo experiments, we assumed that the cameras were fully calibrated prior to running our system. For the RGB-D configuration, depth images were registered to the RGB frame and undistorted using the intrinsic parameters provided by the camera. For the stereo configuration, the intrinsic and extrinsic parameters, including the baseline, were taken directly from the official KITTI calibration files and kept fixed during all experiments. These calibration assumptions ensure consistent reprojection accuracy and directly influence the precision of pose estimation.

We evaluated the localization accuracy and processing time of each frame in the tracking thread using two well-known public datasets: the Technical University of Munich (TUM) RGB-D dataset [27] and the Karlsruhe Institute of Technology and Toyota Technological Institute (KITTI) dataset [28]. We tested both RGB-D and stereo cameras with these datasets. The localization accuracy was compared with that of other open-source SLAM systems. To minimize the nondeterminism inherent in the multithreading system, each sequence was run five times, and the median results were used to determine the accuracy of the estimated trajectory. As shown in Section 4.1, we tested our proposed three-strategy ORB feature adaptive extraction algorithm, which demonstrates that our method achieves higher computational efficiency and accuracy. Additionally, we also conducted tests in a real-world environment to further validate our system’s performance.

### 4.1. Feature Matching

We evaluated the performance of the three-strategy ORB feature adaptive extraction algorithm proposed by us based on the inlier rate and computation time. Since our method is implemented based on the feature extraction algorithm of ORB-SLAM2, we compared it with ORB-SLAM2. Using the TUM dataset for testing, we first set the expected number of feature points as N=800, the number of pyramid layers as L=8, and the pyramid scale factor as s=1.2. We obtained matched pairs using brute force matching and then used the RANSAC algorithm to remove outliers and obtain inliers [30].

The comparison of feature matching results between ORB-SLAM2 and our method is shown in Table 2. The quantitative results are shown in Table 3, where “Matches” refers to the matched pairs obtained by brute force matching, “Inliers” refers to the matched pairs after screening, “Ratio” refers to the ratio of “Inliers” to “Matches”, and “Time” refers to the time consumed by feature extraction. The results show that our method achieves higher accuracy than ORB-SLAM2 while reducing computational time. As can be seen from Table 2, even after removing most mismatches through the RANSAC algorithm, there are still some mismatches in the feature extraction method of ORB-SLAM2, while our method has almost no mismatches.

### 4.2. Localization Accuracy and Efficiency Experiments

We evaluated the proposed method based on localization accuracy and frontend odometry tracking time. The absolute pose error (APE) was used to measure the error between the estimated trajectory from the system and the ground truth, calculated as follows:

(23) $$APE_{RMSE}=\sqrt{\frac{1}{n}\sum_{i=1}^{n}{\left({\bar{X}}_{i}-X_{i}\right)}^{2}}$$

where ${\bar{X}}_{i}$ is the estimated pose from the system at time i, and $X_{i}$ is the ground truth pose. A lower APE indicates higher localization accuracy.

#### 4.2.1. TUM RGB-D Dataset

The TUM dataset consists of indoor sequences captured by RGB-D cameras, including various lighting, structural, and textural conditions. Since our proposed method is implemented based on ORB-SLAM2, we compared it with ORB-SLAM2 and other representative visual SLAM systems in terms of average per-frame frontend odometry tracking time and trajectory error comparison, as shown in Table 4 and Table 5, respectively.

In Table 4, our method exhibits marginally higher RMSE in sequences like fr1/desk1 and fr1/desk2. This stems from repetitive textures and weak features in these scenes, causing brief optical flow mismatches. To preserve real-time performance, we restricted feature matching and local BA to keyframes, yielding sparser constraints than full feature-based or dense optimization methods, thus reducing drift suppression in texture-limited scenarios.

**Figure 5:**
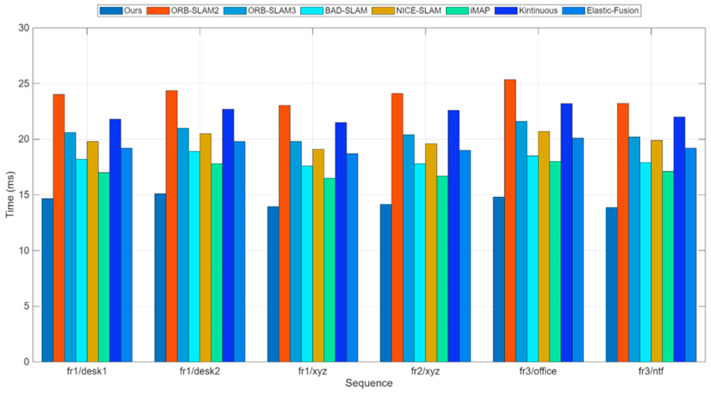
Time consumption comparison for RGB-D on the TUM dataset. Average runtime comparison of all six sequences for different methods; more details are in Table 4.

The experimental results indicate that both our method and ORB-SLAM2 exhibit high robustness. Notably, our approach extracts features and descriptors exclusively for keyframes, leading to nearly a twofold improvement in computational efficiency. Furthermore, in the fr3/ntf sequences characterized by vigorous camera motion, our method achieves higher localization accuracy compared to other state-of-the-art visual SLAM systems. Figure 5 shows the comparison of per-frame processing time in the frontend tracking thread for the fr1/desk1 sequence.

**Figure 6:**
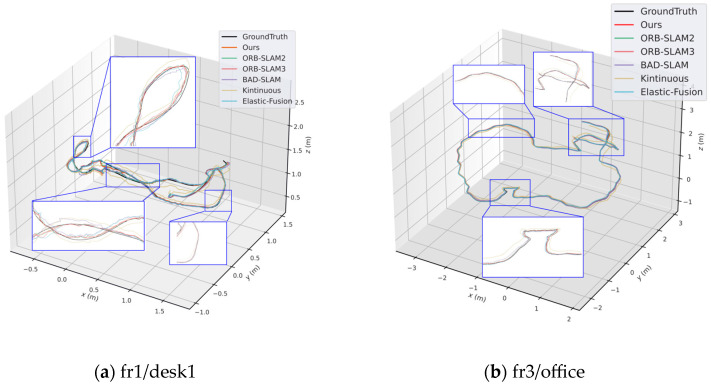
Localization accuracy comparison on the TUM dataset. (**a**) results on the fr1/desk1 sequence, (**b**) results on the fr3/office sequence.

Due to the improvements we made to the feature extraction algorithm in ORB-SLAM2, the stability and efficiency of feature extraction are enhanced. Therefore, the keyframe processing time of our method is slightly lower than ORB-SLAM2. Moreover, using the pose predicted by the uniform motion model as the initial value for optical flow tracking effectively reduces the iteration time of the multilayer optical flow method. Figure 5 shows the comparison of average per-frame processing time between our method and ORB-SLAM2 on six sequences from the TUM dataset. Figure 6 shows the trajectory and error comparison on the fr1/desk1 and fr3/office sequences.

All experiments were conducted on a workstation equipped with an Intel Core i7 CPU (Intel Corporation, Santa Clara, CA, USA) and an NVIDIA GeForce RTX 3080 GPU (NVIDIA Corporation, Santa Clara, CA, USA). The system was compiled using g++ with -O3 optimization, and all methods were executed under identical hardware and compilation settings to ensure a fair comparison.

**Figure 7:**
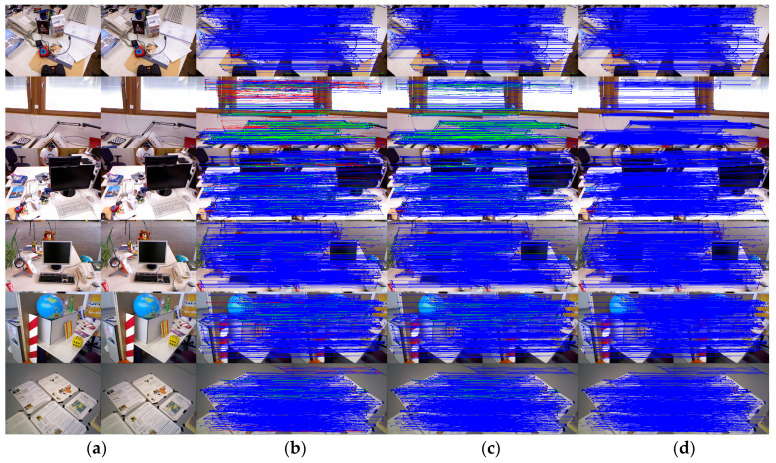
Keypoint tracking examples of the system on the TUM dataset, with 1000 features extracted per frame. The first to sixth rows show keypoint tracking examples on the TUM dataset. The left and right images in (**a**) represent the reference and current frame, respectively (image pairs). (**b**) The multilayer optical flow tracking result based on the uniform motion model (MOF). (**c**) The outlier removal result of reverse optical flow tracking (ROF). (**d**) The outlier removal result based on the fundamental matrix using RANSAC (RANSAC). (Blue represents the final retained feature points after the two-stage outlier removal process, red indicates the outliers eliminated by backward optical flow tracking, green denotes the outliers removed through the RANSAC-based fundamental matrix estimation).

Figure 7 shows the outlier removal results in two adjacent frames of the TUM dataset. Table 6 shows the time required for optical flow tracking and outlier removal. Reverse optical flow tracking takes the multilayer optical flow tracking result as the initial value, which can reduce the number of algorithm iterations and thus improve the running speed. Our method averages 2.04 ms to track an image of size 640 × 480 per frame.

#### 4.2.2. KITTI Stereo Dataset

The KITTI dataset consists of outdoor sequences captured by a stereo camera in urban and highway environments. We compared our method with ORB-SLAM2 and VINS-Fusion [22], a stereo-only and loop closure-enabled system, respectively. Table 7 and Table 8 show the comparison results of frontend odometry tracking time and trajectory errors, respectively.

**Figure 8:**
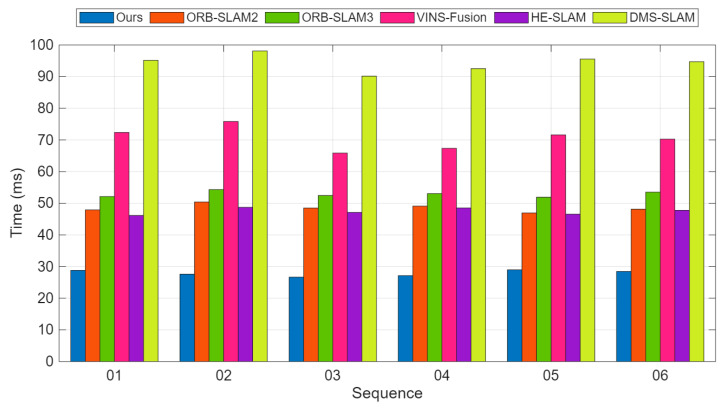
Time consumption comparison for stereo on the KITTI dataset. Average runtime comparison of all six sequences for different methods. More details are in Table 7.

**Figure 9:**
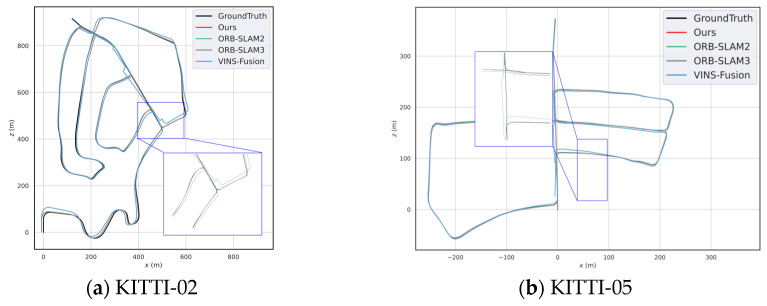
Trajectory comparison on sequence (**a**) results on the KITTI-02 sequence and (**b**) results on the KITTI-05 sequence.

Our method is capable of loop detection and re-localization in loop closure scenarios. In the KITTI-01 sequence, there are more dynamic obstacles and larger disparity changes when the vehicle turns, so our localization accuracy is relatively lower on this sequence. However, the tracking thread speed of our method is significantly better than ORB-SLAM2. Figure 8 shows the comparison of per-frame tracking time in the frontend thread on the KITTI-01 sequence. Figure 8 shows the average per-frame processing time comparison between our method and ORB-SLAM2 on six KITTI sequences. Figure 9 illustrates the trajectories of different methods on 02 and 05.

Our method achieved trajectory accuracy comparable to ORB-SLAM2 while demonstrating significantly higher localization precision than VINS-Fusion in both sequences.

**Figure 10:**
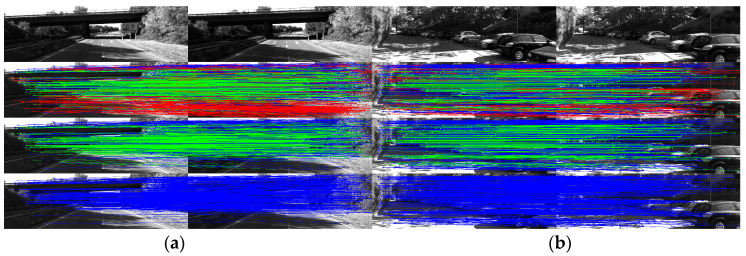
Keypoint tracking examples on the KITTI dataset. (**a**) Scene 1; (**b**) Scene 2. Each group of images from top to bottom shows the reference and current frame (image pairs), the multilayer optical flow–tracking result based on a uniform motion model (MOF), the outlier-removal result of reverse optical flow tracking (ROF), and the outlier-removal result based on the fundamental matrix using RANSAC. Blue represents the final retained feature points after the two-stage outlier-removal process; red indicates the outliers eliminated by backward optical-flow tracking; green denotes the outliers removed through the RANSAC-based fundamental matrix estimation.

Figure 10 shows the outlier removal results in two adjacent frames of the KITTI dataset. Table 9 shows the time required for optical flow tracking and outlier removal. Our method averages 3.97 ms to track an image of size 1226 × 370 per frame on the KITTI dataset.

As shown in Table 8, the proposed method exhibits a noticeable performance degradation on KITTI sequences 01 and 02 compared to other sequences. This is primarily due to the nature of these sequences, which involve high-speed driving scenarios with frequent sharp turns and the presence of dynamic objects such as cars and pedestrians. Under such conditions, the assumptions of the optical flow method—namely, small inter-frame motion and photometric consistency—tend to break down. Rapid rotational motion can cause large pixel displacements between consecutive frames, which challenges the accuracy of sparse optical flow tracking. In addition, dynamic objects introduce inconsistent motion patterns, making it difficult to establish stable correspondences, especially when no semantic filtering is applied.

These results highlight the limitations of our method in highly dynamic or rapidly changing environments. In future work, we aim to address this by integrating IMU data for improved pose prediction and incorporating semantic segmentation modules to suppress the influence of dynamic elements in the scene.

#### 4.2.3. Real-World Experiments

**Figure 11:**
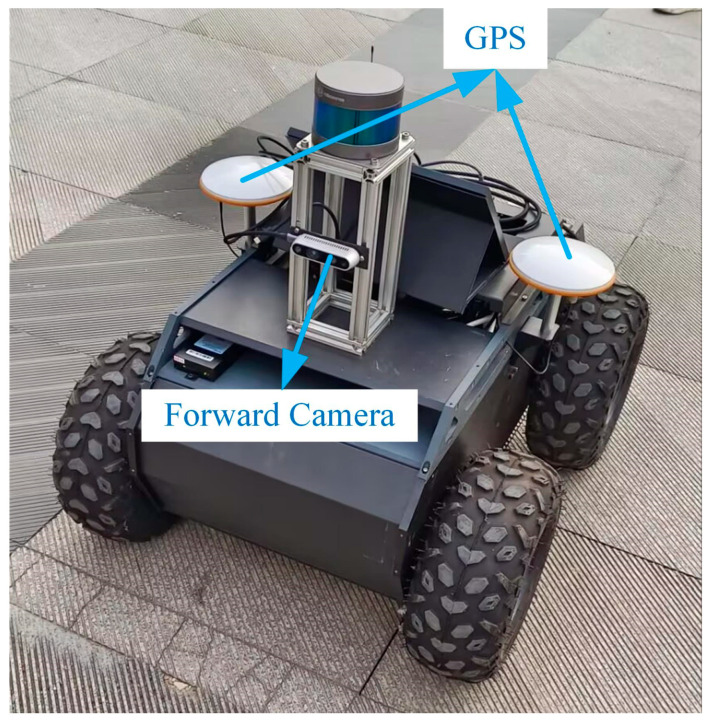
The mobile robot is equipped with a RealSense depth camera (1280 × 720 resolution) and a GPS. The depth camera captures image frame data, while the GPS data serves as the ground truth for the robot’s pose.

To further validate the effectiveness of the proposed method, we conducted tests in a real-world environment. The mobile robot used in the experiment is shown in Figure 11 and is equipped with an Intel RealSense D435 depth camera (Intel Corporation, Santa Clara, CA, USA), a RoboSense LiDAR (RoboSense, Shenzhen, China). In this experiment, we only utilized the camera and GPS, where the camera was used to capture image frames, and the GPS data served as the reference ground truth.

To ensure accurate synchronization between the camera and GPS data, we adopted a timestamp-based synchronization approach. Each image frame and corresponding GPS reading was assigned a precise timestamp, and the data were temporally aligned using interpolation to match each camera frame with its corresponding GPS position. In cases where the time deviation exceeded a defined threshold, a temporal windowing strategy was employed to discard misaligned data, thereby guaranteeing high-quality input. We selected absolute pose error (APE) as the primary evaluation metric, as it quantitatively measures the accuracy of the estimated trajectory with respect to ground truth. By computing the deviation between the estimated and reference poses, APE offers an intuitive and reliable indicator of localization accuracy, particularly in real-world scenarios. This metric is widely adopted in SLAM systems, especially in dynamic environments, due to its effectiveness in assessing trajectory consistency across different motion patterns. In addition, we also computed the root mean square error (RMSE) to evaluate the system’s positional stability and consistency over time. Experiments were conducted across various real-world environments, encompassing both loop and non-loop scenarios, to comprehensively assess the robustness and accuracy of our system under dynamic and complex conditions.

**Figure 12:**
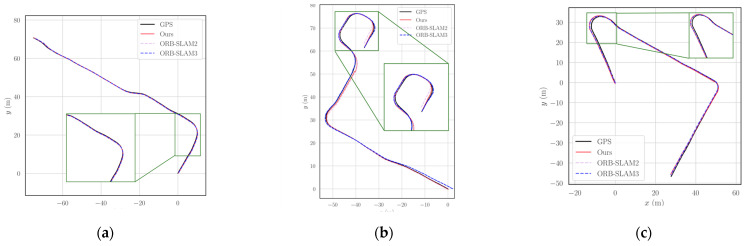

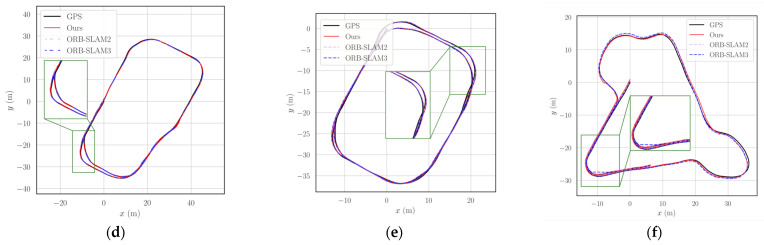
Comparison of the trajectories between our method and ORB-SLAM2 in real-world scenarios. (**a**) st1; (**b**) st2; (**c**) st3; (**d**) st4; (**e**) st5; (**f**) st6. Each subplot illustrates the GPS ground truth, the trajectories estimated by ORB-SLAM2 and ORB-SLAM3, and the trajectories obtained by our method.

We conducted six experiments in different environments, including both loop and non-loop scenarios, and compared the localization results of our method with ORB-SLAM2, as summarized in Table 10 and Table 11. The results for each sequence are presented as the median values across five executions, where RMSE denotes the translational error in meters, and time represents the average per-frame tracking time in milliseconds. The experimental results indicate that the computation time of our method is significantly lower than that of ORB-SLAM2. In the st2 and st5 sequences, our method achieves higher localization accuracy than ORB-SLAM2, and it also demonstrates strong competitiveness in other sequences. The comparison of localization trajectories for all sequences is shown in Figure 12.

**Figure 13:**
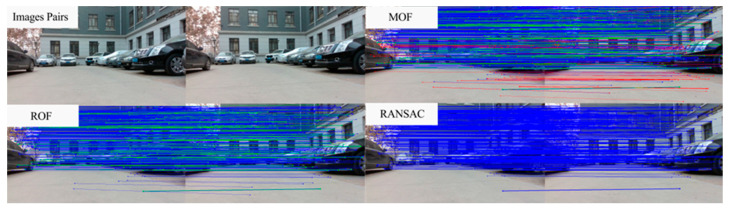
Examples of keypoint tracking in a real-world environment are presented, with 2000 features extracted per frame. (Blue represents the final retained feature points after the two-stage outlier removal process, red indicates the outliers eliminated by backward optical flow tracking, green denotes the outliers removed through the RANSAC-based fundamental matrix estimation).

Figure 13 illustrates the outlier removal results for two consecutive frames in a real-world environment. Table 12 provides the computational details, showing that our method achieves an average processing time of 3.85 ms per frame for 1280 × 720 images, highlighting its efficiency in optical flow tracking and outlier removal.

## 5. Conclusions

We propose a general lightweight visual SLAM approach for RGB-D and stereo cameras: GL-VSLAM. GL-VSLAM uses a multi-level optical flow method based on the uniform motion model prediction to replace feature matching based on descriptors, significantly improving running efficiency. To ensure the positioning accuracy of the system, a coarse-to-fine strategy is introduced to improve the accuracy of pose estimation. The initial value of the camera pose is calculated by PnP based on the robust keypoint correspondences obtained by sparse optical flow, and a further refined pose is obtained by minimizing reprojection error. Our method balances the competing needs between localization accuracy and computational complexity. Qualitative and quantitative results show that our method achieves state-of-the-art accuracy and faster speed performance, which is almost two times that of ORB-SLAM2. However, the optical-flow-based tracking still relies on the assumptions of photometric consistency and small inter-frame motion, and its robustness may degrade in the presence of rapid rotations, significant motion blur, or abrupt illumination changes. Furthermore, without semantic or motion segmentation, the system remains sensitive to dynamic objects that introduce inconsistent motion patterns and reduce the stability of optical flow correspondences. These factors limit performance in highly dynamic or rapidly changing environments and motivate further enhancements to improve robustness.

In future work, we plan to further integrate an IMU and LiDAR to mitigate the limitations of a single sensor and enhance robustness in scenarios involving rapid motion.

## Figures and Tables

**Table 1:** Hyperparameter settings used in our method.

Category	Parameter	Symbol	Value
Pyramid	Pyramid levels	L	8
Pyramid	Pyramid scale factor	s	1.2
Feature Count	Expected feature count	$N_{k}$	1000
Region Division	Region division factor	k	0.8
Thresholding	Global extraction coefficient	ε	5/10
Thresholding	Minimum threshold	δ	5
Quadtree	Maximum quadtree depth	$D_{max}$	3–5

**Table 2:** Comparison of feature matching results.

	Image Pairs	ORB-SLAM2	Ours
1	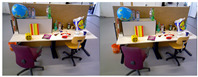	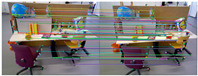	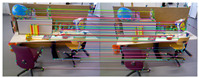
2	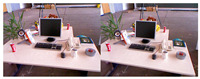	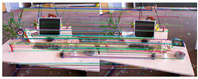	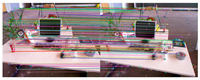
3	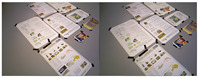	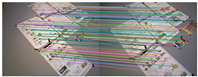	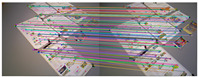
4	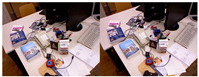	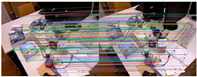	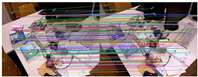
5	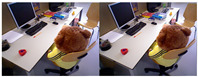	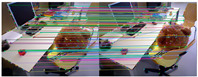	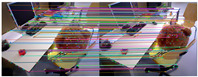
6	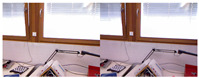	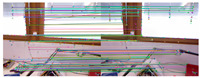	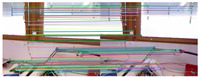

**Table 3:** Feature matching comparison of ratio (%) and time (ms).

Sequence	ORB-SLAM2			Ours		
	Inliers/Matches	Ratio	Time	Inliers/Matches	Ratio	Time
1	209/327	0.64	16.58	216/323	0.67	13.56
2	201/303	0.66	17.24	216/309	0.70	13.35
3	308/372	0.83	18.34	327/381	0.86	13.81

**Table 4:** Average time (ms) comparison on the TUM dataset.

	fr1/desk1	fr1/desk2	fr1/xyz	fr2/xyz	fr3/office	fr3/ntf
Ours	14.66	15.11	13.94	14.15	14.82	13.87
ORB-SLAM2 [8]	24.02	24.37	23.04	24.11	25.35	23.21
ORB-SLAM3 [20]	20.60	21.09	19.82	20.42	21.64	20.26
BAD-SLAM [11]	18.23	18.92	17.68	17.82	18.53	17.96
NICE-SLAM [24]	19.86	20.54	19.12	19.65	20.72	19.93
iMAP [23]	17.02	17.86	16.54	16.78	18.01	17.16
Kintinuous [9]	21.81	22.74	21.56	22.61	23.24	22.04
Elastic-Fusion [10]	19.26	19.84	18.65	19.06	20.14	19.21

**Table 5:** RMSE (m) comparison on the TUM dataset; “–” means that we could not obtain the value from the literature.

	fr1/desk1	fr1/desk2	fr1/xyz	fr2/xyz	fr3/office	fr3/ntf
Ours	0.018	0.026	0.009	0.007	0.018	0.037
ORB-SLAM2 [8]	0.015	0.023	0.014	0.005	0.022	0.048
ORB-SLAM3 [20]	0.016	0.025	0.012	0.004	0.016	0.044
BAD-SLAM [11]	0.028	0.032	0.018	0.011	0.023	0.040
NICE-SLAM [24]	0.027	–	–	0.018	0.030	–
iMAP [23]	0.049	–	–	0.020	0.058	–
Kintinuous [9]	0.046	0.075	0.021	0.033	0.049	0.36
Elastic-Fusion [10]	0.025	0.056	0.012	0.009	0.028	0.32

**Table 6:** Time (ms) of the multi-level optical flow method and outlier removal on the TUM dataset.

	fr1/desk1	fr1/desk2	fr1/xyz	fr2/xyz	fr3/office	fr3/ntf	Average
MOF	1.26	1.25	1.23	1.18	1.24	1.21	1.23
ROF	0.66	0.72	0.77	0.69	0.71	0.74	0.72
RANSAC	0.08	0.10	0.08	0.09	0.08	0.11	0.09
Total	2.00	2.07	2.08	1.96	2.03	2.06	2.04

**Table 7:** Average time (ms) comparison on the KITTI dataset.

	01	02	03	04	05	06
Ours	28.81	27.60	26.66	27.13	28.97	28.46
ORB-SLAM2 [8]	47.91	50.41	48.51	49.10	46.95	48.13
ORB-SLAM3 [20]	52.12	54.32	52.45	53.06	51.91	53.51
VINS-Fusion [22]	72.36	75.84	65.86	67.35	71.61	70.25
HE-SLAM [31]	46.14	48.72	47.11	48.53	46.56	47.78
DMS-SLAM [32]	95.15	98.12	90.16	92.53	95.56	94.72

**Table 8:** RMSE (m) comparison on the KITTI dataset.

	01	02	03	04	05	06
Ours	10.04	5.41	0.28	0.15	0.62	0.77
ORB-SLAM2 [8]	9.43	4.96	0.36	0.19	0.38	0.65
ORB-SLAM3 [20]	8.29	4.53	0.33	0.18	0.51	1.20
VINS-Fusion [22]	6.55	18.41	0.87	0.21	4.43	2.63
HE-SLAM [31]	10.22	5.74	0.99	0.23	0.81	0.812
DMS-SLAM [32]	9.70	6.03	0.52	0.24	0.76	0.64

**Table 9:** Time (ms) of the multi-level optical flow method and outlier removal on the KITTI dataset.

	01	02	03	04	05	06	Average
MOF	2.61	2.58	2.49	2.59	2.41	2.56	2.54
ROF	1.41	1.26	1.28	1.37	1.34	1.38	1.34
RANSAC	0.11	0.09	0.08	0.08	0.09	0.10	0.09
Total	4.13	3.93	3.85	4.04	3.84	4.04	3.97

**Table 10:** Average runtime (ms) comparison on real-world dataset.

	st1 (w/o Loop)	st2 (w/o Loop)	st3 (w/o Loop)	st4 (w/o Loop)	st5 (w/o Loop)	st6 (w/o Loop)
Ours	29.67	31.46	30.86	33.06	32.08	34.75
ORB-SLAM2 [8]	42.47	41.07	42.51	45.84	46.98	48.24
ORB-SLAM3 [20]	53.68	61.93	60.84	64.73	57.45	65.28
VINS-Fusion [22]	72.36	74.53	75.68	76.40	79.48	73.69

**Table 11:** RMSE (m) comparison on real-world dataset.

	st1 (w/o loop)	st2 (w/o loop)	st3 (w/o loop)	st4 (w/o loop)	st5 (w/o loop)	st6 (w/o loop)
Ours	0.25	0.51	0.70	0.39	0.30	0.57
ORB-SLAM2 [8]	0.31	0.71	0.54	0.37	0.32	0.45
ORB-SLAM3 [20]	0.28	0.65	0.44	0.36	0.34	0.49
VINS-Fusion [22]	0.26	1.14	0.97	0.44	0.64	0.81

**Table 12:** Processing time (ms) of the multi-level optical flow method and outlier removal in a real-world environment.

	str1	st2	st3	st4	st5	st6	Average
MOF	2.35	2.48	2.69	2.62	2.71	2.27	2.52
ROF	1.21	1.30	1.29	1.28	1.22	1.21	1.25
RANSAC	0.07	0.08	0.07	0.07	0.08	0.09	0.08
Total	3.63	3.86	4.05	3.97	4.01	3.57	3.85

## Data Availability

The original data presented in the study are openly available in public repositories. The TUM RGB-D dataset is available at https://vision.in.tum.de/data/datasets/rgbd-dataset (accessed on 10 August 2025). The KITTI Odometry dataset is available at https://www.cvlibs.net/datasets/kitti (accessed on 10 August 2025). The original contributions presented in this study are included in the article. Further inquiries can be directed to the corresponding authors.

## Abbreviations

Abbreviations	English full form
APE	Absolute Pose Error
BA	Bundle Adjustment
BRIEF	Binary Robust Independent Elementary Features
DSO	Direct Sparse Odometry
FAST	Features from Accelerated Segment Test
GPS	Global Positioning System
IMU	Inertial Measurement Unit
LiDAR	Light Detection And Ranging
MLP	Multilayer Perceptron
MOF	Multi-level Optical Flow
ORB	Oriented FAST and Rotated BRIEF
OTSU	Otsu Thresholding Method
PnP	Perspective-n-Point
RANSAC	Random Sample Consensus
RMS	Root Mean Square
RMSE	Root Mean Square Error
ROF	Reverse Optical Flow Tracking
SLAM	Simultaneous Localization And Mapping
SVO	Semi-Direct Visual Odometry
VINS-Mono	Visual–Inertial Navigation System—Mono
VINS-Fusion	Visual–Inertial Navigation System—Fusion
VSLAM	Visual Simultaneous Localization and Mapping
